# Immunomodulatory Effect of COVID-19 on HLA-Antibody Profile in Renal Transplant Recipients

**DOI:** 10.3390/jcm13082383

**Published:** 2024-04-19

**Authors:** Marina Kljajic, Zoran Sabljic, Ivana Juric, Vesna Furic Cunko, Renata Zunec, Marija Burek Kamenaric, Bojan Jelakovic, Nikolina Basic-Jukic

**Affiliations:** 1 Department of Nephrology, Arterial Hypertension, Dialysis and Transplantation, University Hospital Centre Zagreb, 10000 Zagreb, Croatia; 2 Tissue Typing Centre, Clinical Department of Transfusion Medicine and Transplantation Biology, University Hospital Centre Zagreb, 10000 Zagreb, Croatia

**Keywords:** transplantation, HLA, immunomodulation, SARS-CoV-2, COVID-19

## Abstract

**Background/Objectives:** The novel coronavirus disease 2019 (COVID-19) has led to significant morbidity and mortality among kidney transplant recipients. SARS-CoV-2 has been hypothesized to cause an unusual immunological dysregulation triggering alloimmunity and leading to graft rejection. **Methods:** This prospective observational cohort study assessed 321 kidney transplant recipients who had COVID-19 infection. After the infection, patients’ sera were tested for the presence of anti-HLA de novo DSA and non-DSA specificities. Logistic regression analysis and a stepwise multivariable logistic regression analysis were used to analyze the independent risk factors associated with the development of antibodies, adjusting for known confounders. The variables evaluated were acute COVID-19 characteristics (i.e., presentation, and need for hospitalization), demographic characteristics (i.e., age, gender, and primary renal disease), clinical characteristics (i.e., various comorbidities), and post-COVID-19 sequelae. **Results:** Anti-HLA de novo DSA developed in 18.7% of patients, while anti-HLA class I and class II non-DSA antibodies developed de novo in 84 (26.3%) and 83 (25.9%) patients, respectively. The development of DSA, HLA-DQ, and HLA-DR antibodies was predicted by the history of graft rejection. Obesity appeared to be protective against the emergence of de novo DSA. De novo DSA and HLA-DR antibody formation was positively linked with intravenous immunoglobulin use, CMV-hyperimmune globulin use, and decreased doses of immunosuppression during acute infection. Better allograft function during the acute disease was a protective factor against the formation of HLA-DQ and HLA-DR antibodies. Positive predictors of de novo DSA development were graft biopsy and the reactivation of EBV after infection. **Conclusions:** These findings suggest that the SARS-CoV-2 virus has an immunomodulatory effect and may be associated with an increased mortality in this population.

## 1. Introduction

Human leukocyte antigens (HLAs) are genes in major histocompatibility complexes (MHCs) that encode proteins responsible for regulating our immune system and distinguishing between “own” and “foreign” antigens. In accordance with the functions and characteristics of their genetic products, HLAs are separated into three regions on the short arm of chromosome 6 and categorized as class I, class II, and class III [1]. Class I HLAs are present on the surface of almost all nucleated cells and are involved in the presentation of endogenous peptides to responding CD8+ T cells. On the other hand, HLA class II molecules are restricted to antigen-presenting cells and involved in exogenous peptide presentation to CD4+ helper T cells [1]. Antibodies against human leukocyte antigens (anti-HLAs) are produced after exposure to different tissue during an organ transplantation, pregnancy, or blood transfusions [1]. They can be divided into class I and class II anti-HLAs. Anti-HLA donor-specific antibodies (DSAs), directed specifically against the donor’s HLA antigens, have been demonstrated to be an important risk factor for graft rejection or loss in renal transplantation [2]. It is advisable to frequently check the anti-HLA antibody profiles of kidney transplant recipients [3]. The majority of anti-HLA antibodies develops 6 months after the transplantation. Class I anti-HLA antibodies are usually the first ones to appear, followed by Class II [4]. Understanding the mechanism of HLA antibody-mediated graft damage is essential given the close correlation between the presence of HLA antibodies and reduced transplant function and survival. If anti-HLA DSAs develop after the transplantation, their binding to donor cells activates the complement cascade and promotes neutrophil infiltration, which leads to microvascular injury and, consequently, graft dysfunction and failure [5]. This antibody-mediated acute rejection is characterized by immunologic evidence of renal injury and evidence of circulating anti-HLA DSAs [6]. In addition to the features of acute rejection, cellular proliferation, apoptosis, and the development of vascular lesions also occur in the setting of chronic rejection due to intracellular signaling triggered by the agonistic activation of HLA I, HLA II, endothelial, or epithelial cell surface markers [5]. Allograft dysfunction has been linked to several viral infections, including the Epstein–Barr virus (EBV), Herpes Simplex, Varicella Zoster, and Cytomegalovirus (CMV). It has been postulated that these infections stimulate the development of anti-HLA antibodies via T-cell cross-reactivity [7,8,9]. The novel coronavirus disease 2019 (COVID-19) brought on by the SARS-CoV-2 virus has had a huge impact on the kidney transplant population. Due to their immunosuppressive regimen, transplant recipients are more vulnerable to viral infections and have a higher mortality rate [9]. They have had serious outcomes (hospitalization, intensive care unit admission, and death) from COVID-19–related disease both during the acute infection and in the post-COVID follow-up [10,11,12]. Moreover, many transplantation surgeries were delayed or cancelled, which impacted the management and care of wait-listed patients [13]. The increased mortality from COVID-19 disease among kidney transplant recipients is explained by a higher incidence of concomitantly present illnesses such as diabetes mellitus, hypertension, iatrogenic-induced immunosuppression, and cardiovascular diseases, which are all known risk factors for the development of a severe form of the disease [13]. Chronic immunosuppressive therapy decreases T- and B-cell function and further contributes to the worse outcomes in the transplant population [13]. Kidney graft rejection following COVID-19 disease has been described in the literature [14,15,16]. Vasquez-Jimenez et al. [14] performed kidney graft biopsies in 20 kidney transplant patients 4 weeks after recovery from COVID-19 disease and demonstrated histological signs of graft rejection among 14 (70%) patients. It has been postulated that COVID-19, caused by severe acute respiratory syndrome coronavirus 2 (SARS-CoV-2), causes unusual immunologic dysregulation and triggers allosensitivity in kidney transplant recipients by the formation of HLA de novo DSA and non-DSA specificities. 

The aim of this study was to evaluate factors affecting the development of de novo DSA and HLA antibodies after acute COVID-19 infection in kidney transplant recipients. 

## 2. Materials and Methods

A prospective observational cohort study evaluated outcomes of 321 kidney transplant recipients after the initial diagnosis of COVID-19 disease. 

### 2.1. Study Subjects 

**Figure 1:**
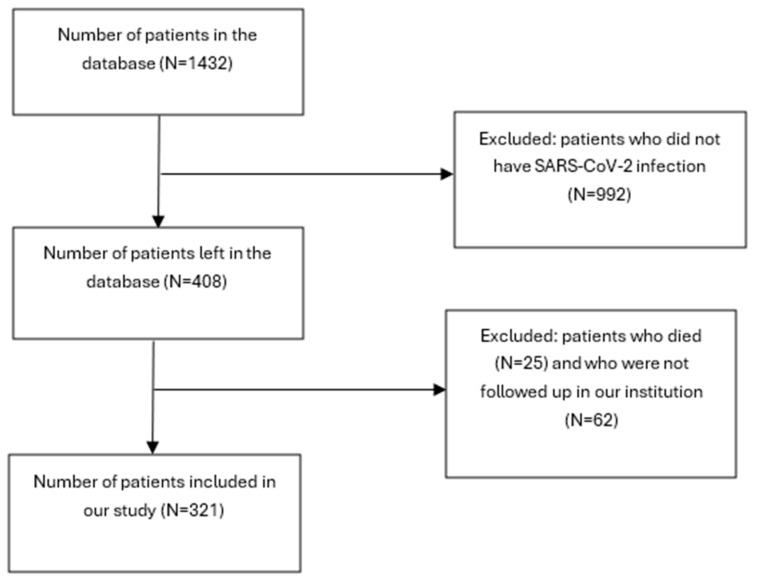
Flow-chart of the study population.

From March 2020 to December 2022, 408 out of the initial cohort of 1432 patients who received renal allograft at Clinical Hospital Centre Zagreb developed COVID-19 disease, proven by positive SARS-CoV-2 real-time reverse transcriptase polymerase chain reaction (RT-PCR) on the nasopharyngeal swab, and were potentially eligible for investigation. Twenty-five patients died in the period during or after the infection and 62 patients have not been assessed in our clinic and were, therefore, excluded from the study population (Figure 1).

Patient characteristics prior to SARS-CoV-2 infections (demographics, comorbidities, primary kidney disease, maintenance immunosuppression regimen, history of graft rejection, vaccination, and HLA class I and class II mismatches (MM) prior to transplantation) were obtained from the hospital database and are described in Results. 

### 2.2. Data Collection

Data on treatment during the acute COVID-19 infection, changes in immunosuppressive regimen, and acute COVID-19 characteristics were obtained from the medical records. Data were modified after being taken out of the electronic medical record to be suitable for statistical analysis. There was no substitution for the missing data.

The initial assessment at the outpatient clinic was performed three months after the acute SARS-CoV-2 infection and included following procedures:

- To assess clinical complications, patients were interviewed by a standardized survey by trained transplant nephrologists to recount symptoms during the acute illness and whether they persisted or some new ones occurred to assess clinical complications. They also underwent a detailed physical examination.
- Venous blood samples were collected for complete blood count, biochemistry, coagulation examinations (prothrombin time (PT), activated partial thromboplastin time (APTT) and fibrinogen), D-dimers, C3, C4, total complement, platelet aggregation with ADP (adenosine 5′-diphosphate), and serum electrophoresis. By means of polymerase chain reaction, patients’ sera were tested for Cytomegalovirus (CMV), Epstein–Barr Virus (EBV), and BK Virus (BKV) infections. Allograft dysfunction was defined as the new onset increase in serum creatinine by 25% or by newly developed proteinuria.
- Both LABScreen™ Mixed Class I and II (One Lambda, Canoga Park, LA, USA) commercial test and LIFECODES LSA Class I and Class II Single Antigens test (Werfen/Immucor Transplant Diagnostics Inc., Stamford, CT, USA) were used for detection of DSA and anti-HLA antibodies by Luminex bead-based technology. Patient’s sera were first tested with a LABScreen™ Mixed test. If the test showed positive reaction for the presence of HLA class I and/or HLA class II antibodies, further testing with LIFECODES single antigen test was performed. Mean fluorescence intensity (MFI) values ≥ 1000 were considered positive. Results were compared with historical values from the database obtained prior to COVID-19.

### 2.3. Statistical Analysis

The primary outcomes included the de novo development of DSA and anti-HLA antibodies. Categorical data were presented by absolute and relative frequencies. The normality of the distribution of continuous variables was tested by the Shapiro–Wilk test. Continuous data were described by the median and the limits of the interquartile range (IQR). The Mann–Whitney U test was used to compare the median between the two groups, while Fisher’s exact test was used to analyze the differences between proportions. Logistic regression analysis was used to analyze the independent factors associated with the development of de novo DSA and HLA antibodies. A stepwise multivariable logistic regression was used to assess the association between potential risk factors and the development of de novo DSA and HLA antibodies after COVID-19, adjusting for known confounders. Variables assessed included demographic characteristics (i.e., age, gender, and primary kidney disease), clinical characteristics (i.e., different comorbidities), acute COVID-19 characteristics (i.e., presentation, and need for hospitalization), and post-COVID-19 complications. Parameters with statistical significance in the univariate analysis were incorporated into the multivariate logistic regression model for in-depth analysis. The level of significance was set at an alpha of 0.05. Considering the relatively small sample size and the possibility of overfitting in the multivariate logistic regression model, we adopted a forward stepwise method (probability for stepwise: entry P 0.1) for logistic regression analysis to reduce the number of independent variables entering the model. There was no substitution of the missing data. The statistical analysis was performed using MedCalc^®^ Statistical Software version 19.6 (MedCalc Software Ltd., Ostend, Belgium; https://www.medcalc.org (accessed on 26 March 2023)) and the IBM SPSS Stat. 23 (IBM Corp. Released 2015. IBM SPSS Statistics for Windows, Version 23.0. IBM Corp., Armonk, NY, USA).

## 3. Results

### 3.1. Study Population (Patients’ Characteristics)

Most frequent causes of death reported in COVID-positive patients were sepsis (19 patients), acute respiratory insufficiency (4 patients), and acute myocardial infarction (3 patients). Sepsis and acute respiratory insufficiency were reported together as causes of death in 3 patients, while sepsis and acute myocardial infarction were reported together in 1 patient. There was a slight prevalence of male patients in the studied population (57%). According to BMI calculations prior to SARS-CoV-2 infection, there were 105 (32.8%) patients who were of normal weight, compared to 4 (1.3%), 144 (45%), and 67 (20.9%) patients who were underweight, pre-obese, and obese, respectively. Forty-six patients (14.4%) had a history of allograft rejection; and 46,6% of patients received at least one dose of the anti-SARS-CoV-2 vaccine before the infection (Table 1).

One hundred twenty-five (39.1%) patients required hospitalization, 141 (44.1%) developed pneumonia, and 4 patients (1.3%) required mechanical ventilation. Treatment included immunosuppression modification in 233 patients (77.1%) and remdesivir in 53 patients (16.6%). CMV-hyperimmune globulin (Cytotect) was introduced in 30 patients (9.4%) and 13 (4.4%) patients received intravenous immunoglobulins (IVIg). In the short follow-up period after COVID-19 disease, one or more rehospitalizations were necessary for 70 patients (21.9%). Biopsy was performed in 25 (7.8%) patients due to a worsening of the allograft function. After SARS-CoV-2 infection, CMV, BKV, and EBV reactivations were confirmed in 72 (22.5%), 61 (19.1%), and 115 (35.9%) patients, respectively (positive urine and/or blood PCR test). SARS-CoV-2 reinfection was confirmed in 78 (25.2%) patients.

### 3.2. Analysis of DSA and HLA Antibodies

Class I HLA antibodies developed de novo in 84 (26.3%) patients, while class II antibodies developed de novo in 83 (25.9%) patients. We have noticed a higher prevalence of certain class II antibodies among the patients which we have shown in Table 1. There was an increase in incidence of HLA-DR (especially HLA-DR53), HLA-DP, and HLA-DQ (especially HLA-DQB1*06, HLA-DQ7, HLA-DQ8, and HLA-DQ9). HLA-DR antibodies developed in 45 (14.1%) patients, while HLA-DP and HLA-DQ developed in 4 (1.3%) and 63 (19.7%) patients, respectively. Seventeen (5.3%) patients developed HLA-DR53 antibodies, 31 (9.7%) patients HLA-DQ8, 28 (8.8%) patients developed HLA-DQ9, and 26 (8.1%) of them HLA-DQ7 antibodies. Donor-specific antibodies (DSA) developed de novo in 57 (18.7%) patients (Table 2).

### 3.3. Analysis of Predictors for De Novo DSA Development

The bivariate analysis identified 10 significant predictors for the development of de novo DSA after SARS-CoV-2 infection (Table 2). The strongest predictors for DSA development were previous graft rejection and the female sex, while a higher BMI and vaccination after COVID-19 decreased the probability of DSA development. Furthermore, higher initial immunosuppression maintenance doses of prednisolone, adjustment of immunosuppressive therapy (decreasing Tacrolimus/Cyclosporin A dose), use of CMV immunoglobulins (Cytotect), and IVIg during the infection were also proven to be statistically significant predictors for de novo DSA development. From the post-COVID-19 period, a performed biopsy was a strong predictor of concurrent de novo DSA development. EBV reactivation also appeared to have a positive correlation with de novo formed DSA. Stepwise multivariate regression analysis was used to further examine significant predictors for de novo DSA development. Two predictors (female sex (OR = 2.75) and previous graft rejection (OR = 5.97)) had a unique statistically significant contribution to the model. They are significant predictors that increase the probability of DSA development, while obesity decreases the probability (OR = 0.24). The model was completely statistically significant (X^2^ = 26.9; *p* < 0.001) and explained from 13% (according to Cox and Snell R2) to 22% (according to Negelkerke R2) of the variance in the presence of DSA (Table 3). 

### 3.4. Analysis of Predictors for De Novo HLA DQ and HLA DR Antibody Development

The bivariate analysis identified five statistically significant predictors for the development of de novo HLA DQ antibodies post-COVID-19 (Table 3). The strongest predictor for de novo HLA DQ antibody development was previous graft rejection. A higher prednisolone maintenance dose and performed biopsy were also positive predictors. A better allograft function estimated by the Chronic Kidney Disease Epidemiology Collaboration equation (CKD-EPI) during and post SARS-CoV-2 infection, decreased the probability of de novo HLA DQ antibody development. Stepwise multivariate regression analysis showed that patients with BMI within the normal range (OR = 2.24) and those with previous graft rejection (OR = 3.84) had an increased probability for de novo HLA DQ development, while the better allograft function estimated by the CKD EPI values (during COVID-19 infection) decreases the probability (OR = 0.97). The model was completely statistically significant (X^2^ = 31.1; *p* < 0.001) and explained from 10% (according to Cox and Snell R2) to 16% (according to Negelkerke R2) of the variance in the presence of HLA DQ (de novo) (Table 4).

The bivariate analysis identified four statistically significant predictors for the development of de novo HLA DR antibodies after COVID-19 disease (Table 4). The strongest protective effect was exhibited by the better allograft function estimated by the CKD-EPI value during SARS-CoV-2 infection and at the post-COVID check-up. Previous graft rejection and IV Ig application increased the probability of de novo HLA DR antibody development. Furthermore, the multivariate statistical analysis confirmed that previous graft rejection (OR = 3.84) is a significant factor that increases the probability of de novo HLA DR antibody development, while the better allograft function estimated by the CKD EPI values (during COVID-19 infection) decreases the probability (OR = 0.97). The model was completely statistically significant (X^2^ = 17.4; *p* < 0.001) and explained from 7% (according to Cox and Snell R2) to 13% (according to Negelkerke R2) of the variance in the presence of HLA DR (de novo) (Table 5).

## 4. Discussion

To our knowledge, this is the first study to compare the likelihood of development of DSA and specific HLA antibodies after COVID-19 infection in renal transplant recipients considering their demographic characteristics (i.e., age, gender, and primary kidney disease), clinical characteristics (i.e., different comorbidities), acute COVID-19 characteristics (i.e., presentation, and need for hospitalization), and post-COVID-19 complications. De novo DSA developed in 57 out of 305 (18.7%) patients, which is less than the percentage obtained in the study from Girnita AL et al. [17] where 14 of 46 (30%) patients developed DSA, and more than in the study from Masset C et al. [18] where the global incidence of post-COVID-19 DSA was 4% (7 out of 179 patients). According to our results, patients who developed de novo DSA were more likely to be female. A history of graft rejection appeared to be a common predictive factor for the development of DSA, HLA DQ, and HLA DR, which could be explained by an increased predisposition to the alloimmune response of those patients. Interestingly, obesity appeared to be a protective factor against the development of de novo DSA. The presence of obesity in transplanted patients reduced the likelihood of de novo DSA development after SARS-CoV-2 infection, whereas a normal body weight was associated with an increased prevalence of newly developed HLA-DQ antibodies. A possible explanation could be that obese patients were in a more immunosuppressed state. Obesity has a detrimental effect on immunity and affects leukocyte populations [19,20]. Higher rates of vaccination failure and infection-related consequences serve as evidence for this [19,21]. A study from Killian JT et al. [22] found that patients with new or increased DSA levels were less likely to have received at least one dose of a COVID-19 vaccination prior to infection (0% vs. 28%, *p* = 0.018), while our bivariate analysis found vaccination after COVID-19 infection to be a protective factor against de novo DSA development. The COVID-19 and/or SARS-CoV-2 mRNA vaccines have also been found to neither increase nor cause the development of new anti-HLA antibodies [23]. Furthermore, the results of our bivariate analysis showed that a higher pre-admission immunosuppressant dose of prednisolone increased the likelihood of both de novo DSA and HLA DQ antibody development, which is in accordance with the results from Killian JT et al. [22]. Their study found that patients with new or increased DSA levels had higher pre-admission immunosuppressant doses, which is consistent with more recent transplantation and stronger immunosuppression. Our bivariate analysis showed that lowering the doses of maintenance immunosuppression (decreasing the dose of Tacrolimus/Cyclosporin A) during the time of acute SARS-CoV-2 infection positively correlated with the probability of de novo DSA development. Moreover, treatment with IVIg and CMV-hyperimmune globulin (Cytotect) increased the likelihood of de novo DSA and HLA DR development. There is evidence that the “cytokine storm”, a disproportionately high level of inflammation and activation of the immune system, contributes to the severity of COVID-19 disease [24]. Our hypothesis is that patients with severe COVID-19 disease had a stronger activation of the immune system, which predisposed them to the development of anti-HLA antibodies. At our hospital, the modulation of immunosuppressive therapy and treatment with IVIg and Cytotect were initiated in patients with more severe forms of COVID-19 disease. The intravenous administration of IVIg and Cytotect was initiated due to their immunomodulatory effects and potential antiviral effects against SARS-CoV-2, which has been associated with favorable outcomes in kidney transplant patients with COVID-19 disease [25,26]. On the other hand, patients with milder forms of the disease lacked a strong systemic inflammatory response that would induce acute kidney injury through a cytokine storm, endothelial dysfunction, hypoxic injury, and hypercoagulability [27]. Consequently, kidney allografts worked better in the milder forms of the disease. The development of anti-HLA antibodies is, therefore, not directly related to a better allograft function during the disease but indicates a link between milder forms of the disease, the weaker activation of the immune system, and, consequently, the smaller chance of anti-HLA antibody development. Similar to our study, Killian et al. found an increased DSA response to be associated with an impaired allograft function [22]. Two positive predictors of de novo DSA development, namely, graft biopsy (performed in patients with a worsening renal function) and the reactivation of EBV after SARS-CoV-2 infection, suggest an immunomodulatory effect of the SARS-CoV-2 virus. COVID-19 infection can lead to the reactivation of latent viruses such as EBV through an impairment of the immune system and lymphopenia [28]. Co-infection with Epstein–Barr virus (EBV) has been associated with severe clinical cases of COVID-19 infection as well as symptoms of long COVID [28,29]. Stefanelli et al. [28] analyzed EBV DNA six months before and after SARS-CoV-2 infection. Of the 166 patients with COVID-19 who completed the study, 50% showed the reactivation of EBV DNA. A higher EBV reactivation was observed in older patients with more severe COVID-19 cases and in patients who required hospitalization. In our study, HLA class I antibodies were slightly more prevalent (84/236 (26.3%)) than class II (83/237 (25.9%)) in de novo developed HLA antibodies post-COVID-19 infection which is contrary to the results obtained in the study from Girnita AL et al. where more anti-HLA antibodies were predominantly directed against HLA Class II (20/26, 77%) [5]. Girnita AL et al. also found that most DSAs targeted HLADQ (71%), with a dominant IgG isotype and IgG1 subtype prevalence (93%), and/or IgG3 (64%), followed by IgG2 (36%). The most prevalent type of de novo class II HLA antibodies in our study was HLA DQ which developed in 63 (19.7%) patients. 

There are a few limitations of this study. Firstly, the recovery from acute COVID-19 dictated the visitation schedule. This meant that each patient’s check-up did not occur at the same time, which could influence the outcomes. Moreover, the lack of baseline information on HLA and DSA antibodies before COVID-19 disease may have led to the overestimation of de novo developed HLA and DSA antibodies. Besides that, the absence of baseline data on the EBV DNA expression may have caused an overestimation of EBV reactivations. Additionally, we lacked information on all patients’ intrahospital laboratory results and medical care during the acute COVID-19 since not all of them were treated in our institution. Finally, this was a single-center study carried out in a tertiary referral facility. This may potentially restrict the generalizability of our findings along with the study’s limited sample size. We also recognize that the observed correlations are not conclusive and do not prove a causal relationship. On the other hand, this study is one of the first studies to specifically address the issue of post-COVID-19 immunogenicity in renal transplant recipients. It covered both hospitalized and outpatient patients, providing crucial insights into clinical issues that might arise even in individuals who initially appear with no symptoms.

## 5. Conclusions

In conclusion, more research is urgently needed since these findings point to an immunomodulatory effect of the SARS-CoV-2 virus and may be linked to serious clinical consequences such as acute graft rejections and increased mortality in kidney transplant recipients. Although no such research in the general population has been made, SARS-CoV-2 immunogenicity may also be an issue affecting a broader population. A greater emphasis should be given to transplant recipients’ post-COVID-19 clinical assessment, especially regarding de novo DSA and HLA antibody development, to help guide actions to avert serious complications and mortality. All COVID-19 survivors should be observed for a longer period to assess any newly arising issues and treat them. Further research with long-term follow-up is required.

## Figures and Tables

**Table 1:** Patients’ characteristics.

	Number (%) of Patients	Min–Max
Sex		
M	183 (57.2)	
F	137 (42.8)	
Age (years) [Median (IQR)]	55 (44–64)	22–81
Primary kidney disease		
Glomerulonephritis	98 (30.6)	
DM	12 (3.8)	
Polycystic kidney disease	48 (15)	
Pyelonephritis	26 (8.1)	
Nephroangiosclerosis	26 (8.1)	
Other	110 (34.4)	
Time since transplantation (months) [Median (IQR)]	94.5 (52–135.8)	1–368
Height (cm) [Median (IQR)]	171 (163–180)	124–199
Body mass (kg) [Median (IQR)]	79 (67–92)	42–150
BMI [Median (IQR)]	26.5 (23.9–29.2)	17.36–45.79
Nutritional status		
Underweight (<18.5)	4 (1.3)	
Normal weight	105 (32.8)	
Pre-obese (25–29.9)	144 (45)	
Obese (≥30)	67 (20.9)	
History of graft rejection	46 (14.4)	
Immunosuppression		
Tacrolimus	222 (69.4)	
Cyclosporin A	70 (21.9)	
Mycophenolate	280 (87.5)	
Azathioprine	12 (3.8)	
Everolimus	48 (15)	
Corticosteroid dose (mg) [Median (IQR)]	5 (5–5)	0–30
Creatinine [Median (IQR)]	129 (98–165.8)	45–430
CKD EPI [Median (IQR)]	49 (35–64)	0.23–133
Vaccinated against COVID-19	246 (76.9)	
Before COVID-19 disease	149 (46.6)	
After COVID-19 disease	97 (30.3)	
Number of applied vaccine doses (*n* = 246)		
One	21 (8.5)	
Two	138 (56.1)	
Three	83 (33.7)	
Four	4 (1.6)	

M—male, F—female, DM—Diabetes mellitus, BMI—Body mass index.

**Table 2:** De novo developed DSA and HLA antibodies after SARS-CoV-2 infection.

	Number (%) of Patients
HLA class I	
De novo	84 (26.3)
HLA class II	
De novo	83 (25.9)
HLA-DR	
De novo	45 (14.1)
HLA-DP	
De novo	4 (1.3)
HLA-DQ	
De novo	63 (19.7)
HLA-DQB1*06	
De novo	4 (1.3)
HLA-DR53	
De novo	17 (5.3)
HLA-DQ8	
De novo	31 (9.7)
HLA-DQ9	
De novo	28 (8.8)
HLA-DQ7	
De novo	26 (8.1)
DSA	
De novo	57 (18.7)

**Table 3:** Bivariate and multivariate analysis used to examine predictors of de novo DSA development.

**Bivariate Analysis**	**ß**	***p***	**OR**	**95% CI**
Sex (F)	0.87	0.004	2.38	1.32–4.29
Previous graft rejection	1.84	<0.001	6.30	3.17–12.5
Vaccination after COVID-19 infection	−0.83	0.03	0.43	0.20–0.93
BMI	−0.09	0.007	0.91	0.84–0.97
Prednisolone dose	0.10	0.002	1.11	1.04–1.18
COVID-19				
Decreasing Tacrolimus/Cyclosporin A	0.96	0.03	2.62	1.09–6.29
Cytotect	1.02	0.02	2.76	1.19–6.42
Intravenous Immunoglobulins	1.32	0.04	3.73	1.09–12.72
Post-COVID-19				
Biopsy	1.20	0.009	3.33	1.35–8.24
EBV	0.73	0.01	2.08	1.16–3.75
**Multivariate Analysis**	**ß**	***p***	**OR**	**95% CI**
Sex (F)	1.16	0.006	3.20	1.39–7.37
Previous graft rejection	1.79	<0.001	5.97	2.35–15.17
Nutritional status (obesity)	−1.41	0.03	0.24	0.07–0.89
Constant	−2.21	<0.001		

ß—coefficient of regression.

**Table 4:** Bivariate and multivariate analysis used to examine predictors of de novo HLA DQ antibody development.

**Bivariate Analysis**	**ß**	***p***	**OR**	**95% CI**
Previous graft rejection	1.58	<0.001	4.87	2.49–9.52
Prednisolone dose	0.08	0.006	1.09	1.02–1.16
COVID-19				
CKD EPI	−0.02	0.002	0.98	0.96–0.98
Post-COVID-19				
CKD EPI	−0.02	0.008	0.98	0.97–0.99
Biopsy	0.93	0.04	2.54	1.06–6.07
**Multivariate Analysis**	**ß**	***p***	**OR**	**95% CI**
Nutritional status (normal weight)	0.81	0.01	2.24	1.19–4.21
Previous graft rejection	1.35	<0.001	3.84	1.83–8.05
CKD EPI—post-COVID-19	−0.02	0.01	0.97	0.96–0.98
Constant	−1.02	0.02		

ß—coefficient of regression.

**Table 5:** Bivariate and multivariate analysis used to examine predictors of de novo HLA DR antibody development.

**Bivariate Analysis**	**ß**	***p***	**OR**	**95% CI**
Previous graft rejection	1.08	0.004	2.95	1.41–6.19
COVID-19				
CKD EPI	−0.02	0.004	0.98	0.96–0.99
Intravenous immunoglobulins	1.43	0.02	4.19	1.3–13.5
Post-COVID-19				
CKD EPI	−0.03	0.001	0.97	0.96–0.99
**Multivariate Analysis**	**ß**	***p***	**OR**	**95% CI**
Previous graft rejection	1.35	0.003	3.84	1.58–9.36
CKD EPI—post-COVID-19	−0.02	0.04	0.97	0.96–0.98
Constant	−1.03	0.04		

ß—coefficient of regression.

## Data Availability

The raw data supporting the conclusions of this article will be made available by the authors upon request.
